# A Cytoplasmic NAD(P)H-Dependent Polysulfide Reductase with Thiosulfate Reductase Activity from the Hyperthermophilic Bacterium Thermotoga maritima

**DOI:** 10.1128/spectrum.00436-22

**Published:** 2022-06-28

**Authors:** Jiyu Liang, Haiyan Huang, Yubo Wang, Lexin Li, Jihong Yi, Shuning Wang

**Affiliations:** a State Key Laboratory of Microbial Technology, Microbial Technology Institute, Shandong University, Qingdao, People’s Republic of China; b Department of Pathogen Biology, School of Basic Medical Sciences, Shandong First Medical University & Shandong Academy of Medical Science s, Jinan, People’s Republic of China; University of Minnesota

**Keywords:** elemental sulfur reduction, thiosulfate reduction, sulfur reductase, NAD(P)H-dependent polysulfide reductase, *Thermotoga maritima*, thermophile

## Abstract

Thermotoga maritima is an anaerobic hyperthermophilic bacterium that efficiently produces H_2_ by fermenting carbohydrates. High concentration of H_2_ inhibits the growth of T. maritima, and S^0^ could eliminate the inhibition and stimulate the growth through its reduction. The mechanism of T. maritima sulfur reduction, however, has not been fully understood. Herein, based on its similarity with archaeal NAD(P)H-dependent sulfur reductases (NSR), the ORF THEMA_RS02810 was identified and expressed in Escherichia coli, and the recombinant protein was characterized. The purified flavoprotein possessed NAD(P)H-dependent S^0^ reductase activity (1.3 U/mg for NADH and 0.8 U/mg for NADPH), polysulfide reductase activity (0.32 U/mg for NADH and 0.35 U/mg for NADPH), and thiosulfate reductase activity (2.3 U/mg for NADH and 2.5 U/mg for NADPH), which increased 3~4-folds by coenzyme A stimulation. Quantitative RT-PCR analysis showed that *nsr* was upregulated together with the *mbx*, *yeeE*, and *rnf* genes when the strain grew in S^0^- or thiosulfate-containing medium. The mechanism for sulfur reduction in T. maritima was discussed, which may affect the redox balance and energy metabolism of T. maritima. Genome search revealed that NSR homolog is widely distributed in thermophilic bacteria and archaea, implying its important role in the sulfur cycle of geothermal environments.

**IMPORTANCE** The reduction of S^0^ and thiosulfate is essential in the sulfur cycle of geothermal environments, in which thermophiles play an important role. Despite previous research on some sulfur reductases of thermophilic archaea, the mechanism of sulfur reduction in thermophilic bacteria is still not clearly understood. Herein, we confirmed the presence of a cytoplasmic NAD(P)H-dependent polysulfide reductase (NSR) from the hyperthermophile T. maritima, with S^0^, polysulfide, and thiosulfate reduction activities, in contrast to other sulfur reductases. When grown in S^0^- or thiosulfate-containing medium, its expression was upregulated. And the putative membrane-bound MBX and Rnf may also play a role in the metabolism, which might influence the redox balance and energy metabolism of T. maritima. This is distinct from the mechanism of sulfur reduction in mesophiles such as Wolinella succinogenes. NSR homologs are widely distributed among heterotrophic thermophiles, suggesting that they may be vital in the sulfur cycle in geothermal environments.

## INTRODUCTION

Thermotoga maritima is a strict anaerobic hyperthermophilic bacterium that was originally isolated from geothermally heated marine sediments (1). It is unique in that it belongs to a deep-branching lineage of domain Bacteria and possesses numerous genes found only in Archaea (2). T. maritima has drawn considerable attention for its ability to utilize diverse carbohydrates and for its high hydrogen production (1–4). It can ferment 1 mol glucose to about 2 mol acetate, 2 mol CO_2_, and 4 mol H_2_, which is close to the Thauer limit of hydrogen production (4 mol H_2_ per mole glucose) (1, 5). Previous research found that T. maritima growth was significantly inhibited by high H_2_ concentration, whereas in the presence of elemental sulfur (S^0^) and thiosulfate, production of H_2_ decreased and H_2_S was produced instead (1, 5, 6), suggesting that sulfur reduction might have important effects on the metabolism of T. maritima.

Sulfur reduction is a common physiological feature shared by many bacteria and archaea (7–9). S^0^ can be used as the terminal electron acceptor of the respiratory system in several sulfur-reducing bacteria (7, 8, 10). In Wolinella succinogenes, for example, sulfur reduction is linked to H_2_ oxidation. H_2_ is oxidized by a membrane-bound hydrogenase, and the electrons are transferred to S^0^ by cytochromes and the polysulfide reductase PsrABC. Protons are pumped into the periplasm from the cytoplasm during this process, and ATP is further generated by the electrical H^+^ potential (11, 12). The mechanism of S^0^ reduction in *W. succinogenes* has been known as a model of bacterial sulfur reduction.

T. maritima reportedly reduced S^0^ to hydrogen sulfide (H_2_S) after its isolation (1, 5). In the presence of sulfur, T. maritima growth was stimulated, however, the production of acetate, lactate, and CO_2_ remained unaltered (1). Thus, sulfur reduction was assumed to be irrelevant to energy metabolism, and S^0^ was thought to act as an electron sink to prevent H_2_ accumulation (1, 5, 6). Besides, neither has typical membrane-bound polysulfide reductase been discovered in the genome of T. maritima nor have cytochrome or quinone been found to be required for the electron transport chain in T. maritima (2, 3). Unlike W. succinogenes, T. maritima cannot reduce S^0^ through traditional sulfur respiration; rather, S^0^ reduction occurs through a different mechanism.

Besides sulfur-reducing bacteria, many archaea can also reduce S^0^, such as Pyrococcus furiosus, a hyperthermophilic archaea (13). Like T. maritima, P. furiosus is strictly heterotrophic; it produces H_2_ fermentatively, and its growth is also inhibited by H_2_. Sulfur can also stimulate the growth of P. furiosus under H_2_ pressure. Considering these similar properties, T. maritima and P. furiosus may have similar mechanisms of sulfur metabolism. A coenzyme A (CoA)-dependent NAD(P)H:sulfur oxidoreductase (NSR) and a membrane-bound sulfane reductase (MBS) have been discovered and characterized in P. furiosus (14, 15), representing a novel mechanism of sulfur reduction. However, no sulfur reductase in T. maritima has been reported until now.

In this study, we investigated the sulfur reduction activities in cell extracts of T. maritima; we predicted through bioinformatics analysis that an ORF (THEMA_RS02810, the locus tag in GenBank; its old locus tag in GenBank is TM0379) in its genome can encode a protein homologous to the NSR from P. furiosus. The predicted NSR from T. maritima was characterized to catalyze the NAD(P)H-dependent reduction of S^0^, polysulfide, and thiosulfate through heterologous expression, purification, and biochemical analysis. Reverse transcription PCR (RT-PCR) analysis further confirmed that the NSR, together with a putative membrane-bound sulfur oxidoreductase (MBX), may function in the sulfur reduction of T. maritima. Our genome search shows that the NSR homolog is widely distributed in thermophilic bacteria and archaea, suggesting that it might play an important role in the biological sulfur cycle of geothermal environments.

## RESULTS

### Effects of S^0^ on T. maritima growth.

**FIG 1:**
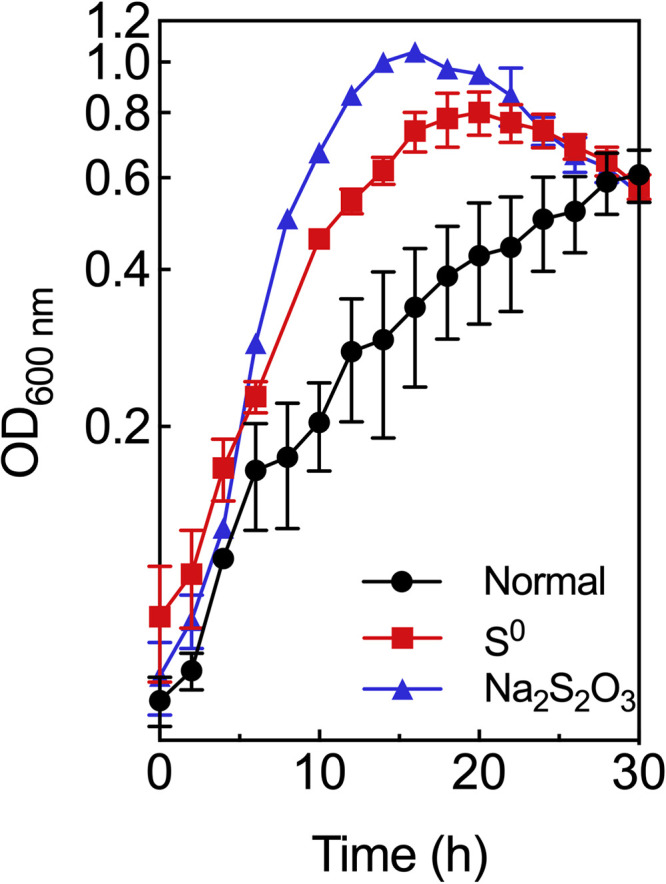
Effects of S^0^ and thiosulfate on T. maritima growth. T. maritima was cultivated in starch medium (black circles), starch medium containing S^0^ (5 g S^0^ powder per L of medium) (red squares), and starch medium containing thiosulfate (30 mM Na_2_S_2_O_3_) (blue triangles).

Unlike other sulfur-reducing bacteria, T. maritima is a heterotrophic bacterium and its growth was not dependent on sulfur respiratory systems. T. maritima grew slowly in the absence of S^0^, reaching an OD_600_ of 0.6 after around 30 h in a starch medium at 80°C (Fig. 1). When cultivated in the starch medium with S^0^, the growth of T. maritima significantly accelerated compared to the growth without S^0^, reaching an OD_600_ of 0.8 after 20 h. Meanwhile, when S^0^ was added to the medium during the growth of T. maritima, H_2_S formation dramatically increased 1 h after sulfur addition (Fig. S1 in the supplemental material).

### Sulfur reductase activity in T. maritima cell extracts.

T. maritima cells cultured in both starch and sulfur-containing media were harvested at the late exponential growth phase to prepare cell extracts by sonication. After a 100,000 × *g* centrifugation, cytoplasmic and membrane fractions were used for sulfur reductase activity assays. No H_2_S production was detected with the membrane fraction, no matter whether NAD(P)H or reduced ferredoxin was used as the electron donor. No ferredoxin-dependent sulfur reductase activity was detected in the cytoplasmic fractions, either. Significant H_2_S production was detected with the cytoplasmic fraction when NAD(P)H was used as the electron donor in the presence of S^0^ (Table 1). NAD(P)H oxidation was also detected by monitoring the absorption decrease at 340 nm (Table 1). The results indicate that the sulfur reduction of T. maritima is an NAD(P)H-dependent reaction and occurs mainly in the cytoplasm. The results also revealed that the NAD(P)H-dependent sulfur reductase activity was dramatically stimulated by CoA (Table 1). CoA-dependent sulfur reductase activity was also observed in thermophilic archaea, such as P. furiosus (14), suggesting that T. maritima may share a mechanism of sulfur reduction similar to P. furiosus. In addition, cell extracts from the S^0^-containing medium presented higher sulfur reductase activities than those from starch medium (Table 1), indicating that S^0^ may promote the expression of the presumed cytoplasmic sulfur reductase.

**TABLE 1:** H_2_S production and NADH oxidation activities in the cytoplasmic fraction of T. maritima cell extracts

Cell extracts	H_2_S production (U/mg)		NADH oxidation (U/mg)	
	NADH + S^0^	NADH + S^0^ + CoA	NADH + S^0^	NADH + S^0^ + CoA
Starch medium (no S^0^)	<0.01	0.12 ± 0.10	0.09 ± 0.04	0.21 ± 0.11
S^0^ medium	0.14 ± 0.08	0.48 ± 0.10	0.40 ± 0.16	0.81 ± 0.25

### Heterologous expression and characterization of the cytoplasmic sulfur reductase NSR of T. maritima.

Attempts to purify the sulfur reductase from T. maritima cell extracts failed because of their poor stability. However, we observed that the sulfur reductase reaction of T. maritima was stimulated by CoA, which is similar with that of the reported cytoplasmic NSR in hyperthermophilic archaea P. furiosus and Thermococcus kodakarensis, implying that the sulfur reductase of T. maritima may be similar to NSR from the two archaea. After searching the T. maritima genome, we found that the protein encoded by the ORF THEMA_RS02810 (its old locus tag is TM0379) contains similar conserved domains (a FAD/NAD binding domain and a FAD/NAD-linked reductase dimerization domain) with NSRs from P. furiosus and T. kodakarensis (Fig. S2 in the supplemental material). It shares 57% identity in protein sequence with the NSR from T. kodakarensis and 34% identity with the NSR from P. furiosus. Besides, sequence analysis indicated that this protein does not have a transmembrane domain or signal peptide. This suggests that THEMA_RS02810 may encode a cytoplasmic enzyme involved in sulfur reduction in T. maritima.

**FIG 2:**
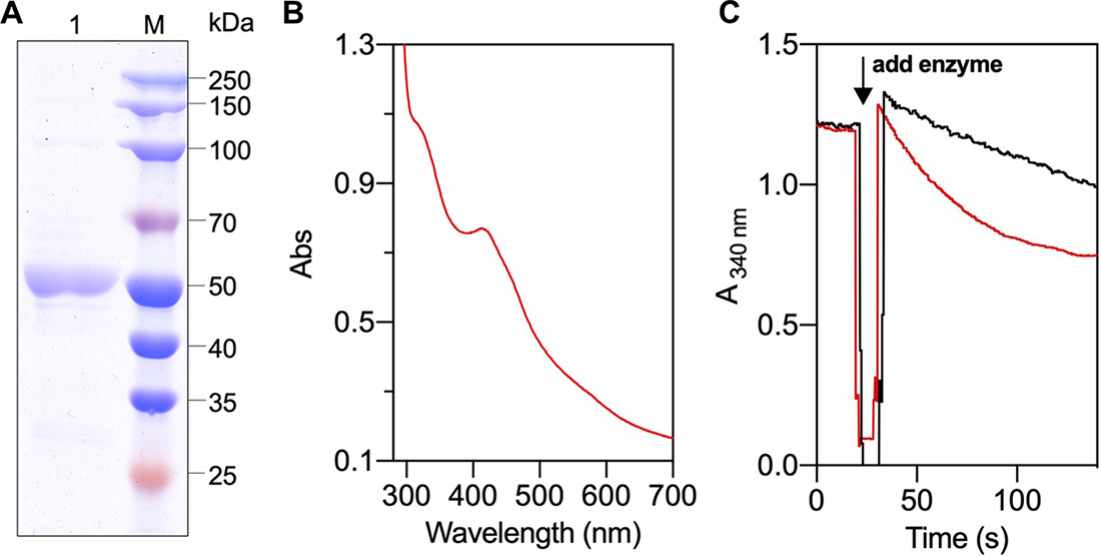
Heterologous expression and characterization of the cytoplasmic sulfur reductase NSR of T. maritima. (A) SDS-PAGE of recombinant NSR. (B) UV–vis absorption of recombinant NSR. (C) NADH oxidation with S^0^ by recombinant NSR. Activities of NSR were measured with the addition of CoA (red curve) and without CoA (black curve). NADH oxidation was observed with the decrease of absorption at 340 nm; reactions were started by the addition of the enzyme.

THEMA_RS02810 was then heterologously expressed in Escherichia coli strain BL21, and the corresponding His-tagged protein was purified anaerobically. The purified recombinant enzyme had a molecular weight of around 50 kDa, as shown by SDS-PAGE analysis (Fig. 2A). This is consistent with the deduced molecular mass according to its protein sequence. The enzyme is yellow in color and exhibits an UV–visible (UV–vis) spectrum with two peaks at around 320 and 430 nm (Fig. 2B). Thin-layer chromatography analysis showed that it binds FAD as its cofactor (Fig. S3 in the supplemental material). The purified recombinant enzyme presented a specific NAD(P)H: S^0^ reductase activity (Table 2). With S^0^ as the electron acceptor, the recombinant protein had a NADH oxidation activity of 0.8 U/mg and a NADPH oxidation activity of 1.3 U/mg at 45°C. (One unit (1 U) is defined as the oxidation of 1 μmol NAD(P)H per minute or the formation of 1 μmol H_2_S per minute.) Moreover, when CoA was added to the reaction mixture, the activities increased to 3.2 U/mg (NADH, Fig. 2C) and 4.2 U/mg (NADPH), which is consistent with the observations of T. maritima cell extracts. The apparent *K_m_* values of NADH and NADPH were also comparable (around 0.05 mM for NADH and 0.08 mM for NADPH) (Table 2). The protein also presented H_2_S production activities from S^0^ reduction with NAD(P)H. No ferredoxin-related activity was detected with the recombinant enzyme. The results indicate that the recombinant enzyme is an NAD(P)H-dependent sulfur reductase, and CoA can significantly stimulate the reaction.

**TABLE 2:** Activities of recombinant NSR of T. maritima

Substrates	NAD(P)H oxidation (U/mg)^*a*^	H_2_S production (U/mg)	Apparent *K_m_* (μM)^*b*^
NADH + S^0^ NADH + S^0^ + CoA	0.8 ± 0.08 3.2 ± 0.10	0.39 ± 0.27 1.38 ± 0.76	50 ± 6.3 (NADH）
NADPH + S^0^ NADPH + S^0^ + CoA	1.3 ± 0.02 4.2 ± 0.05	0.45 ± 0.20 1.21 ± 0.32	80 ± 21.2 (NADPH)
NADH + Na_2_S_2_O_3_ NADH + Na_2_S_2_O_3_ + CoA	2.5 ± 0.01 6.8 ± 0.04	1.35 ± 0.49 2.79 ± 1.20	10 ± 1.2 (Na_2_S_2_O_3_) 60 ± 10.3 (NADH)
NADPH + Na_2_S_2_O_3_ NADPH + Na_2_S_2_O_3_ + CoA	2.3 ± 0.02 6.7 ± 0.08	1.56 ± 0.31 2.93 ± 0.82	10 ± 3.1 (Na_2_S_2_O_3_) 80 ± 2.5 (NADPH)

a The NAD(P)H oxidation activities were determined by monitoring absorption at 340 nm at 45°C.

b The apparent *K_m_* was determined and calculated by using NAD(P)H oxidation assays.

Several CoA-dependent NAD(P)H:sulfur oxidoreductases have been characterized in thermophilic archaea, in which the TK1481 from T. kodakarensis has the highest (57%) identity in protein sequence to THEMA_RS02810 of T. maritima. Other characterized NSR homologs include PF1186 from P. furiosus (34% identity) and TK1299 from T. kodakarensis (34% identity). The enzyme TK1481 from T. kodakarensis was identified as a NAD(P)H oxidase and a NAD(P)H:polysulfide oxidoreductase, as well as a CoA-independent NAD(P)H:S^0^ oxidoreductase (16, 17). Both PF1186 and TK1299 were identified as NAD(P)H:sulfur oxidoreductases with strictly CoA-dependent activities; additionally, TK1299 had NAD(P)H oxidation activity with O_2_ as electron acceptor, whereas PF1186 had no oxygen-related activities despite the fact that it is oxygen insensitive. The recombinant THEMA_RS02810 in this research presented some properties different from its archaeal homologs. Unlike TK1299 and TK1481, the NSR of T. maritima (THEMA_RS02810) did not show any NAD(P)H oxidation activity with O_2_ as electron acceptor. Besides, the recombinant THEMA_RS02810 was sensitive to O_2_. After exposure to air for 30 min at room temperature, it lost its sulfur-reducing activity. Besides, the NSR of T. maritima was not strictly CoA-dependent. The recombinant NSR showed a lower sulfur reduction activity without CoA, and the activity was enhanced about four folds in the presence of CoA.

When polysulfide was used as electron acceptor, the recombinant NSR showed a NAD(P)H oxidation activity (0.32 ± 0.11 U/mg for NADH and 0.35 ± 0.08 U/mg for NADPH), and this activity was also enhanced by CoA (1.19 ± 0.22 U/mg for NADH and 1.22 ± 0.32 U/mg for NADPH). The activity was only detected at a high concentration of polysulfide (> 1 mM), where 1 M phosphate buffer (pH 7.0) was used in the reaction to balance pH change. And in the same buffer, the recombinant NSR could hardly reduce S^0^. Considering that S^0^ is insoluble in water, and NSR is located in the cytoplasm of T. maritima, S^0^ is not likely a substrate *in vivo*, polysulfide might be a more possible substrate. So, NSR most likely functions as NAD(P)H-dependent polysulfide reductase.

CoA showed a significant stimulation on sulfur reductase activity of NSR. A possible reason is that the thiol group may react with S^0^ or polysulfide. A NSR homolog from P. horikoshii was reported with CoA persulfide and CoA polysulfide reductase activities (18). The NSR of T. maritima is likely a sulfane sulfur reductase, and CoA could enhance its access to substrate. However, other compounds with thiols, such as cysteine and glutathione, showed no influence on its activities. The mechanism of CoA enhancement on NSR still needs further study.

### Thiosulfate reductase activity of the sulfur reductase NSR from T. maritima.

Members of the genus *Thermotoga* can also reduce thiosulfate in addition to S^0^ (19). Thiosulfate enhanced the growth of T. maritima. In the presence of thiosulfate, T. maritima grew faster and reached an OD_600_ of around 1.0 in 16 h (Fig. 1), whereas in the absence of thiosulfate, it only reached an OD_600_ of around 0.6 in 30 h. Meanwhile, the addition of thiosulfate during the growth of T. maritima led to an obvious increase in H_2_S production (Fig. S3). With NADH as electron donors, thiosulfate reductase activity was observed in T. maritima cytoplasmic fractions (Table S1 in the supplemental material), but no thiosulfate reductase activity was detected in the membrane fractions, indicating that thiosulfate reduction occurs in the cytoplasm. Interestingly, NAD(P)H-dependent thiosulfate reductase activities were detected with the purified recombinant NSR (Table 2), and the apparent *K_m_* of thiosulfate was low (around 10 μM). Similar to the S^0^ reductase activity, the thiosulfate reductase activities were enhanced 3-fold by CoA. These results suggest that in contrast to its P. furiosus and T. kodakarensis homologs, the NSR participates in both S^0^ reduction and thiosulfate reduction. Previous studies have shown that P. furiosus and T. kodakarensis could not reduce thiosulfate to H_2_S (20, 21).

### Transcription analysis of sulfur-reduction-related genes.

Quantitative RT-PCR (qRT-PCR) analysis was used to examine the transcription of the T. maritima *nsr* gene before and after T. maritima was exposed to S^0^ or thiosulfate. We discovered that 20 min after adding S^0^ to the culture, the transcription level of the *nsr* gene increased 2.74-fold, and 1 h later, it increased 6.99-fold. These results are consistent with the finding that the sulfur reductase activity of the S^0^-containing culture was significantly higher than that of normal culture (Table 1), and that H_2_S generation dramatically increased following sulfur addition (Fig. S3 in the supplemental material). The results indicate that the *nsr* gene is upregulated in the presence of S^0^, and that NSR might be involved in the sulfur-reduction metabolism. Furthermore, the addition of sodium thiosulfate also boosted the transcription level of the *nsr* gene, which increased 3.18-fold after 20 min and 7.50-fold after 1 h.

**FIG 3:**
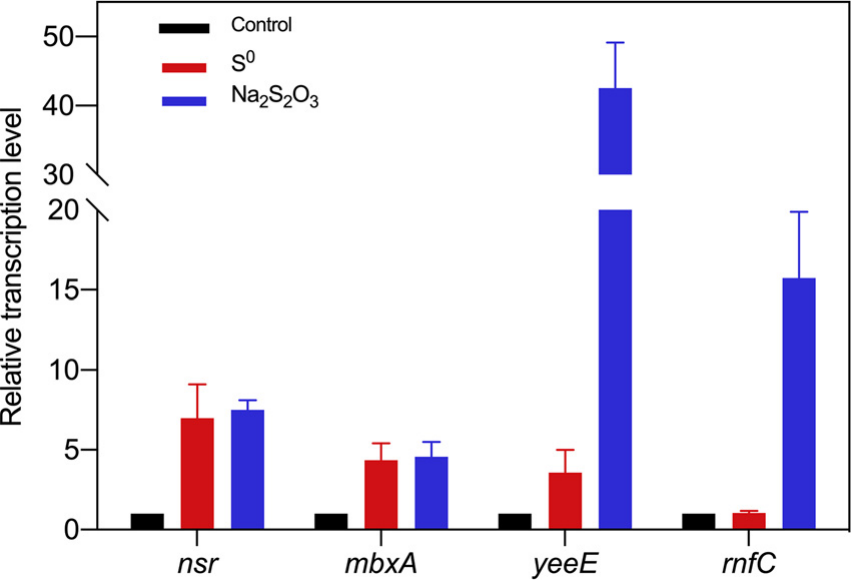
qRT-PCR analysis of T. maritima genes after exposure to S^0^ or thiosulfate. RNA samples were extracted 1 h after the addition of S^0^ and thiosulfate. *nsr* (THEMA_RS02810), NAD(P)H-dependent sulfur reductase; *mbxA* (THEMA_RS08165), subunit A of putative membrane-bound sulfur oxidoreductase; *yeeE* (THEMA_RS09360), a putative thiosulfate transferase; *rnfC* (THEMA_RS03485), subunit C of reduced ferredoxin:NAD^+^ oxidoreductase Rnf.

The transcription levels of other genes were also analyzed by qRT-PCR. Besides the *nsr* gene, *mbxA* (THEMA_RS08165), *yeeE* (THEMA_RS09360), and *rnfC* (THEMA_RS03485) genes were upregulated in the presence of S^0^ or thiosulfate. There were more significant transcription level changes in these genes in the presence of thiosulfate (Fig. 3). The *mbxA* gene is located in a transcriptional unit that encodes a 13-subunit membrane-bound oxidoreductase (MBX), which is a homolog of the MBS (membrane-bound sulfane reductase) complex from P. furiosus. The MBS complex of P. furiosus was recently reported to reduce sulfane to H_2_S and to pump protons to the periplasm using reduced ferredoxin as an electron donor (15). The transcription level of the *mbxA* gene increased 4.36-fold after the addition of S^0^. These results suggest that the MBX complex may also participate in sulfur reduction in T. maritima.

The *yeeE* gene is annotated to encode a membrane protein YeeE, which reportedly mediates thiosulfate uptake in E. coli and Spirochaeta thermophila (22). Interestingly, several *dsrE/tusA* homologs are also located in the same transcription unit, which are involved in thiosulfate transport (23). After exposure to S^0^, the transcription level of *yeeE* increased slightly; when exposed to thiosulfate, however, the transcription level of *yeeE* increased 42.6-fold. Given that T. maritima could reduce thiosulfate in its cytoplasm, the *yeeE*/*dsrE*/*tusA* gene cluster of T. maritima may play an important role in thiosulfate uptake.

RnfC is a subunit of the Rnf complex, a membrane-bound Na^+^-translocating respiratory enzyme that oxidizes reduced ferredoxin and reduces NAD^+^. Rnf has been found to function in the energy metabolism of T. maritima (24). In this study, the transcriptional change of *rnfC* was insignificant after S^0^ addition, whereas in the presence of thiosulfate, the transcription level of *rnfC* was upregulated 15.7-fold, indicating that the Rnf complex plays a key role in energy conservation and balancing reducing equivalents in the presence of thiosulfate. In the presence of S^0^, however, the contribution of the MBX complex might be more important than Rnf for metabolism.

## DISCUSSION

Sulfur is an important element in the lithosphere and biosphere, and microorganisms play a vital role in the sulfur cycle. Sulfate, S^0^, and sulfide are the three most important forms in the sulfur cycle. Sulfate reduction is thought to be a key process in the biological sulfur cycle (7, 25). However, in various geothermal environments where S^0^ is widely distributed, such as deep-sea hydrothermal vents, marine sediments, sulfidic springs, etc., the reduction of S^0^ (vs. sulfate) may be more crucial in the sulfur cycle. Diverse S^0^-reducing microorganisms have frequently been isolated from these hydrothermal habitats (7, 8, 26). Sulfur-reducing prokaryotes are widespread within the phylogenetic tree of life, especially the thermophilic bacteria and archaea, although the metabolism of S^0^ reduction is still not completely understood. The mesophilic bacterium W. succinogenes has been studied as a model organism for sulfur metabolism. The membrane of *W. succinogenes* has an electron transport chain, including a hydrogenase and a molybdenum-bound polysulfide reductase (PsrABC), which oxidizes H_2_ and ultimately transfers electrons to polysulfide. During this process, protons are pumped into the periplasm and subsequently utilized in ATP synthesis via chemiosmosis (27, 28). In some thermophiles, like Acidianus ambivalens and Aquifex aeolicus, membrane sulfur reductase Sre is used for sulfur reduction (29, 30). A group of heterotrophic thermophiles, such as Thermococcous, Pyrococcus, Thermotoga, and Thermoanaerobacter species, also reduce S^0^ to H_2_S. These thermophiles are widely distributed and are evident in the sulfur cycle of geothermal environments, such as hot springs, hot vents, and marine sediments. However, unlike autotrophic sulfur reducers, no typical sulfur reductases like Psr or Sre, have been found in these heterotrophic microbes; neither have any cytochromes nor quinones been found. Therefore, sulfur reduction in these species is not likely accomplished by a respiratory system analogous to *W. succinogenes* or *A. ambivalens*. A different mechanism may contribute to the sulfur reduction in these microbes.

In this study, we report a cytoplasmic NSR from T. maritima that is similar to the NSR of P. furiosus. The NSR of T. maritima catalyzed the NAD(P)H-dependent reduction of S^0^, and transcription of the *nsr* gene increased in the presence of sulfur, suggesting that *nsr* could participate in sulfur reduction. Besides *nsr*, the transcription of *mbxA* increased, suggesting that the MBX complex may be involved in sulfur metabolism. The MBX complex of T. maritima was a homolog of the P. furiosus MBS complex. The MBS complex is thought to be a key enzyme in the sulfur reduction in P. furiosus, which reduces S^0^ with reduced ferredoxin and pumps out H^+^ (15, 31, 32). Thus, we predicted that the MBX complex of T. maritima would also function as a sulfur reductase.

**FIG 4:**
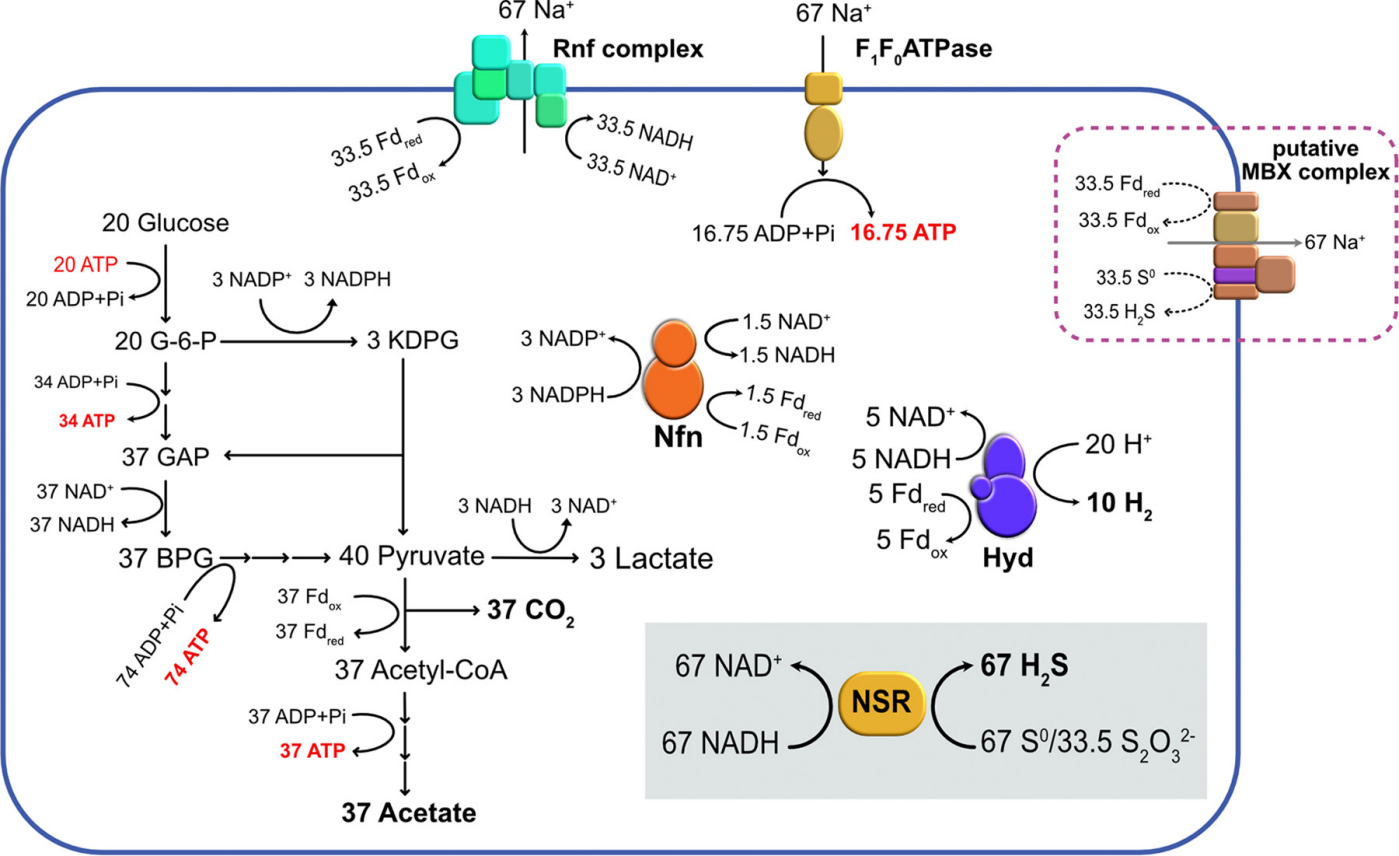
The proposed model for metabolism of T. maritima in the presence of S^0^ or thiosulfate. Nfn, the electron-bifurcating NAD^+^-dependent NADPH:ferredoxin oxidoreductase; Hyd, the electron-bifurcating [FeFe]-hydrogenase; Rnf, reduced ferredoxin:NAD^+^ oxidoreductase; MBX, the putative membrane-bound sulfur oxidoreductase; NSR, NAD(P)H:sulfur oxidoreductase.

Based on these results and on existing knowledge of T. maritima metabolism, we proposed a simplified mechanism of sulfur reduction in T. maritima (Fig. 4), namely, that only glucose is utilized through the EMP and ED pathways, during which NADH, NADPH, and reduced ferredoxin are generated. NADPH can also reduce NAD^+^ and ferredoxin by the electron-bifurcating Nfn (24, 33). Electrons of these reduced equivalents can be further transferred via several pathways: (i) NADH and reduced ferredoxin are oxidized by the electron-bifurcating [FeFe]-hydrogenase (Hyd) to produce H_2_ (34); (ii) NADH or NADPH is used for sulfur reduction with NSR to produce H_2_S; (iii) reduced ferredoxin is used for sulfur reduction with the MBX complex, which is predicted to pump out Na^+^ and generate a Na^+^ potential (Rnf and F_1_F_0_ATPase of T. maritima is Na^+^-dependent (24)); (iv) NADH is used for lactate production (34); and (v) reduced ferredoxin is used to regenerate NADH through the membrane-bound Rnf complex (the Rnf complex in T. maritima is a Na^+^-coupled respiratory reduced ferredoxin:NAD^+^ oxidoreductase) (24).

In the absence of sulfur, the reductants are released mainly by electron-bifurcating Hyd to produce H_2_, as only a small amount of lactate is produced (Fig. S4 in the supplemental material). In the presence of sulfur, the electrons are mainly released through sulfur reduction catalyzed by NSR and MBX instead of H_2_ production (Fig. S5). The transcriptional analysis showed that the increase of *rnfC* gene transcription was not evident after S^0^ addition, whereas *mbxA* and *nsr* were significantly upregulated, indicating that MBX rather than Rnf may play a key role in the presence of S^0^. As more electrons are transferred through the MBX in the process of H_2_S formation than H_2_ formation, more ATP is formed by the Na^+^ potential; therefore, T. maritima grows better in the presence of sulfur. If all the electrons are released by H_2_ production, 0.038 ATP molecules are generated by chemiosmosis per molecule of glucose (Fig. S4), whereas if all the electrons are released by sulfur reduction, 0.96 molecules of ATP per glucose molecule are generated (Fig. S5) (assume that only glucose is utilized). Therefore, T. maritima grows better in the presence of sulfur, especially when the H_2_ pressure is high, and electrons are not released by hydrogenase.

Interestingly, the NSR of T. maritima showed a specific activity of thiosulfate reduction with NAD(P)H that is not found in its archaeal homologs. Thiosulfate is also an important intermediate in the sulfur cycle of anoxic sediments (35), and is involved in sulfur reduction, oxidation, and disproportionation. Thiosulfate reduction is thought to be primarily carried out by sulfate-reducing bacteria in sediments, where thiosulfate functions as an alternative electron acceptor for sulfate respiration. Other bacteria also exhibit thiosulfate reductase activity. The thiosulfate respiratory system has been studied in Salmonella enterica and Pyrobaculum aerophilum, among other species, wherein thiosulfate was reduced by a membrane-bound thiosulfate reductase complex (36, 37). In geothermal environments, thermophiles with thiosulfate reductase activity are widely distributed. For example, researchers view thiosulfate reduction as an important physiological feature of the order Thermotogales (19). However, neither thiosulfate nor a sulfate respiratory system has been found in T. maritima. In this study, the cytoplasmic polysulfide reductase NSR of T. maritima showed significant thiosulfate reductase activity. After the addition of thiosulfate, the *nsr* gene and the putative thiosulfate transferase gene *yeeE* were upregulated; H_2_S was formed dramatically after these genes were expressed. This indicates that thiosulfate reduction may occur in the cytoplasm instead of the membrane, and that NSR may play a role in this metabolism. In the presence of thiosulfate, the *rnfC* gene was significantly upregulated, indicating the involvement of the Rnf complex in the thiosulfate metabolism. Thus, reduced ferredoxin may be oxidized by the Rnf complex for NADH regeneration, and the NADH/NADPH is consumed by NSR to reduce thiosulfate to H_2_S. Similar to the case of S^0^, 0.96 molecules of ATP per glucose molecule is expected to be generated by chemiosmosis if all the electrons are released by thiosulfate reduction (Fig. S6 in the supplemental material) (assume that only glucose is utilized).

A BLAST search was performed to investigate the distribution of NSR in Bacteria and Archaea. Search results revealed that NSR homologs are widely distributed in some groups of bacteria and archaea (Fig. S7 in the supplemental material). Most of these microbes, originally isolated from geothermal environments, are obligate heterotrophic thermophiles with physiological properties similar to T. maritima belonging to three groups: Clostridia, Thermotogales, and Thermococcales. Members of these groups have been frequently reported as sulfur-reducing microbes (19); further investigations confirmed that many of them reduce S^0^ to H_2_S. Some can also reduce thiosulfate (Fig. S7) (7, 38). Caldanaerobacter subterraneus subsp. tengcongensis (previously named Thermoanaerobacter tengchongensis), for example, is a heterotrophic thermophile that can ferment carbohydrates to acetate and reduces S^0^ and thiosulfate; its growth is stimulated by S^0^ and thiosulfate (39). C. subterraneus possesses an NSR homolog (48% identity with the NSR of T. maritima) and contains no typical sulfur respiratory system or MBS complex in its genome, suggesting that NSR may play a key role in the strain’s ability to reduce S^0^ and thiosulfate.

In sum, this study found that the NSR from T. maritima functions as a reductase for S^0^, polysulfide and thiosulfate. The NSR-catalyzed reduction of S^0^ or thiosulfate may significantly affect the strain’s redox balance and energy metabolism, which also involves membrane-bound Rnf and MBX complexes and electron-bifurcating Hyd and Nfn. Considering the wide distribution of the NSR homolog in thermophilic bacteria and archaea and the ubiquitous S^0^/thiosulfate reduction in geothermal environments, NSR may be important in the thermophile-driven sulfur cycle.

## MATERIALS AND METHODS

### Strains and cultivation.

T. maritima (strain DSM 3109) was obtained from DSMZ (German Collection of Microorganisms and Cell Cultures GmbH, Braunschweig, Germany). It was cultured strictly anaerobically at 80°C with 100% gas-phase N_2_ in modified DSMZ medium 1232. The medium was composed of the following (per L): 0.5 g NH_4_Cl; 1.9 g MgSO_4_ × 7H_2_O; 1.6 g K_2_HPO_4_; 1.0 g Na_2_HPO_4_; 26 g NaCl; 10 mL trace element solution; 2.0 g yeast extract; 2.0 g tryptone; 5.0 g soluble starch; 10 mL vitamin solution; and 0.6 g l-cysteine-HCl. Resazurin (0.5 mg/L) was added to the medium as a redox-sensitive indicator. The trace element solution contained the following: 1.5 g nitrilotriacetic acid; 3.0 g MgSO_4_ × 7H_2_O; 0.5 g MnSO_4_ × H_2_O; 1.0 g NaCl; 0.1 g FeSO_4_ × 7H_2_O; 0.18 g CoSO_4_ × 7H_2_O; 0.10 g CaCl_2_; 0.18 g ZnSO_4_ × 7H_2_O; 0.01 g CuSO_4_ × 5H_2_O; 0.01 g H_3_BO_3_; 0.01 g Na_2_MoO_4_ × 2H_2_O; 0.03 g NiCl_2_ × 6H_2_O; 0.3 mg Na_2_SeO_3_ × 5H_2_O; and 0.4 mg Na_2_WO_4_ × 2H_2_O in 1 L distilled water. The vitamin solution contained the following: 2.0 mg biotin; 2.0 mg folic acid; 10.0 mg pyridoxine–HCl; 5.0 mg thiamine–HCl: 5.0 mg riboflavin; 5.0 mg nicotinic acid; 5.0 mg Ca-pantothenate: 0.1 mg vitamin B12; 5.0 mg *p*-aminobenzoic acid; and 5.0 mg lipoid acid in 1 L distilled water. The T. maritima cells were harvested by centrifugation at the late exponential growth phase (OD_600_ ≈ 0.4–0.6) and stored at −80°C until use.

E. coli strains were cultivated in LB or TB medium for heterogeneous expression of specific proteins. The LB medium contained 5 g yeast extract, 10 g tryptone, and 10 g NaCl per L. The TB medium contained 12 g tryptone, 24 g yeast extract, 2.3 g KH_2_PO_4_, 12.5 g K_2_HPO_4_, and 4 mL glycerol.

### Preparation of T. maritima extracts.

T. maritima cells (1 g wet weight) were resuspended with 5 mL of anaerobic 50 mM phosphate buffer (pH 7.4), and 20 mg lysozyme was added. After incubating at 37°C for 6 h, the cells were disrupted by ultrasonication in a vinyl anaerobic chamber (Coy Laboratory Products, Inc.) filled with 95% N_2_ and 5% H_2_. After centrifugation at 10,000 × *g* to remove cell debris, the cell extracts were centrifuged at 100,000 × *g*, and the supernatant and the membrane fraction were used for enzyme assays.

### Heterologous expression and purification of T. maritima sulfur reductase.

A recombinant E. coli BL21 strain was constructed to express the putative sulfur reductase gene (THEMA_RS02810, old_locus_tag: TM0379) of T. maritima. The gene was amplified via PCR with the genomic DNA of T. maritima as a template. The following primers were used: 5′-CGGATCCATGAGGTACGATGTTGTAG-3′ (forward primer, BamHI restriction site underlined) and 5′-CCCTCGAGTAATACCATTTCAGCCG-3′ (reverse primer, XhoI restriction site underlined). After being digested by the restriction enzymes, the PCR product was ligated to the pET-24b (+) plasmid with T4 ligase. The recombinant plasmid was then transferred into E. coli BL21 for heterologous expression.

E. coli BL21-TM0379 was cultivated in TB medium (with 50 mg/L kanamycin); 0.3 mM isopropyl β-D-1-thiogalactopyranoside (IPTG) was added when the OD_600_ reached 0.8. After incubation at 25°C for 16–18 h, the cells were harvested by centrifugation and stored at −20°C for use.

The E. coli BL21-TM0379 cells were disrupted anaerobically in a vinyl anaerobic chamber (Coy Laboratory Products, Inc.) by ultrasonication, and the extracts were centrifuged at 35,000 × *g* for 1 h to remove the debris. The following purification steps were performed anaerobically. The supernatant was water-bathed at 80°C for 30 min and centrifuged at 35,000 × *g*; the supernatant was subsequently pumped into a HisTrap HP column (GE Healthcare) for purification. The column was then washed by 25-, 50-, 75-, 100-, 150-, 250-, and 500-mM imidazole solutions (20 mL for each concentration), and the His-tagged protein was eluted at both 100- and 150-mM imidazole solutions. The purified recombinant protein was concentrated by 30K Amicon Ultra-4 centrifugal filters (Merck Millipore, Ireland), resuspended in 50 mM Tris–HCl (pH 7.4), and stored anaerobically at −80°C for subsequent studies.

### Heterologous expression and purification of T. maritima ferredoxin.

The gene encoding ferredoxin (TM0927) was chemically synthesized and cloned to the pETDuet-1 vector. The recombinant plasmid was transferred to E. coli C41 (with a pRKISC plasmid to enhance iron–sulfur cluster synthesis). The E. coli C41-TM0927 strain was cultured in TB medium (with the addition of 0.2 g/L FeSO_4_, 0.2 g/L ferric citrate, 8 mg/L chloramphenicol, 3 mg/L tetracycline, and 8 mg/L carbenicillin). The expression of ferredoxin was induced by the addition of 0.5 mM IPTG. After incubation at 30°C for 8 h, cells were harvested by centrifugation and stored at −20°C.

E. coli C41-TM0927 cells were disrupted anaerobically by ultrasonication, and debris was removed by 35,000 × *g* centrifugation. The supernatant was water-bathed at 80°C for 30 min to denature the E. coli proteins. After centrifugation at 100,000 × *g* for 1 h, the supernatant was collected. The purity of the heterologously expressed ferredoxin was confirmed by SDS-PAGE and UV-visible absorption analysis (Fig. S8). The purified ferredoxin was stored anaerobically at −80°C until use.

### S^0^ reductase activity assays.

The standard sulfur reductase activity assay mixture (200 μL) contained 50 mM phosphate buffer (pH 7.0), 10 mM NAD(P)H, and 2 mg S^0^. When indicated, 100 μM CoA was added to the mixture. For ferredoxin-related activity, a reduced ferredoxin regeneration system was added to the mixture instead of NAD(P)H. The reduced ferredoxin regeneration system contained 30 μM ferredoxin, 10 U Clostridium pasteurianum hydrogenase (CpI), and 100% gas-phase H_2_ (40). To measure the formation of H_2_S, a modified double-vial technique (14) was used. Gas-phase N_2_ was used, and a 1.5-mL Eppendorf tube was placed in a 10-mL serum bottle and surrounded by 2 mL 1 M NaOH (100% gas-phase H_2_ was used when the reduced ferredoxin regeneration system was used). The reaction mixture was added into the inner tube, and purified enzymes or cell extracts were injected into the inner tube to start the reaction. After incubating at 80°C (a temperature of 40°C was set when the reduced ferredoxin regeneration system was used) for 5 min; the reaction was stopped, and H_2_S was released by the addition of 400 μL sulfuric acid (2 M). The H_2_S was captured by NaOH and its amount measured by methylene blue assay, as described previously (41).

**Thiosulfate reductase activity assays**. The thiosulfate reductase activity assay followed the same procedure as the S^0^ reductase activity assay, except that 40 mM thiosulfate was added as an electron acceptor instead of S^0^. The same double-vial system was used to measure the production of H_2_S.

**NAD(P)H oxidation with S^0^, polysulfide, or thiosulfate**. The oxidation of NAD(P)H was measured in a cuvette (sealed with a rubber stopper), the assay (400 μL) contained 50 mM (1 M was used to stabilize pH when polysulfide as substrate (42)) phosphate buffer (pH 7.0), 0.24 mM NAD(P)H, and 2 mg S^0^, 10 mM sodium polysulfide (prepared as previously described (42)) or 4 mM thiosulfate. When indicated, 100 μM CoA, glutathione or cysteine was added to the mixture, and gas-phase N_2_ was used. Purified enzymes or cell extracts were added to start the reaction. The oxidation of NAD(P)H was monitored by a UV–vis spectrophotometer at 340 nm (ε_340nm_ = 6.2 mM^−1^cm^−1^). The assay was performed at 45°C because it was easier to handle the cuvettes at 45°C than at 80°C (the optimum temperature for T. maritima growth). Most enzymes increase their activity by a factor of 2 for every 10°C rise in the temperature (the Q10 rule).

All solutions, vials, and cuvettes used were prepared or treated anaerobically. One unit (1 U) was defined as the oxidation of 1 μmol NAD(P)H per minute or the formation of 1 μmol H_2_S per minute.

### Quantitative RT-PCR analyses.

T. maritima was cultivated in 100 mL normal starch medium, and 0.5 g S^0^ or 30 mM thiosulfate was added at the middle of its exponential growth phase (OD_600_ ≈ 0.2–0.25). Cells were collected for RNA extraction both before and after (20 min and 1 h) S^0^ or thiosulfate addition. Total RNA of T. maritima was extracted by the *EasyPure* RNA Kit (TransGen Biotech Co., Ltd., Beijing, China) and stored at −80°C until use. cDNA was prepared with an All-In-One 5*×* RT MasterMix (Applied Biological Materials Inc., Canada). The 16S rRNA gene of T. maritima was selected as the reference. The genes *nsr*, *mbxA*, *yeeE*, and *rnfC* were selected for analyses. Primers for these genes in qPCR were designed by Primer-BLAST (https://www.ncbi.nlm.nih.gov/tools/primer-blast/) (Table S2 in the supplemental material). All qPCR experiments were performed with the LightCycler 480 system (F. Hoffmann-La Roche Ltd., Switzerland) using TransStart Top Green qPCR SuperMix (TransGen Biotech Co., Ltd., Beijing, China). The comparative Ct method was used to analyze the results (43).

### Bioinformatic analyses.

Nucleotide and protein sequences were retrieved from the National Center for Biotechnology Information (NCBI) (www.ncbi.nlm.nih.gov/). The T. maritima NSR protein sequence (AAD35465.1) was applied as the query sequence to search for NSR homologs using BLASTp of the NCBI. Protein sequences with identity higher than 40% were selected for further analyses. The neighbor-joining method was used to conduct the multiple sequence alignment and phylogenetic analyses with MEGA 7 (www.megasoftware.net).
