# Supplementary material for: A Cytoplasmic NAD(P)H-Dependent Polysulfide Reductase with Thiosulfate Reductase Activity from the Hyperthermophilic Bacterium Thermotoga maritima
Source: Microbiol Spectr. 2022 Jun 28;10(4):e00436-22. doi: 10.1128/spectrum.00436-22 (PMC9431562; doi:10.1128/spectrum.00436-22)
Supplement: Supplemental file 1 — Supplemental material. Download spectrum.00436-22-s0001.pdf, PDF file, 2.0 MB [file spectrum.00436-22-s0001.pdf]

Supplementary Materials

A Cytoplasmic NAD(P)H-dependent Sulfur Reductase with Thiosulfate Reductase Activity from the Hyperthermophilic Bacterium *Thermotoga maritima*

Jiyu Liang,[a] Haiyan Huang,[b] Yubo Wang,[a] Lexin Li,[a] Jihong Yi,[a] Shuning Wang [a#]

[a]State Key Laboratory of Microbial Technology, Microbial Technology Institute, Shandong University, Qingdao, People's Republic of China

[b]Department of Pathogen Biology, School of Basic Medical Sciences, Shandong First Medical University & Shandong Academy of Medical Sciences, Jinan, People's Republic of China

Running head: A cytoplasmic NSR from thermophilic bacterium

[#] Address correspondence to Shuning Wang, shuningwang@sdu.edu.cn.

Table S1 Thiosulfate reduction activities in *T. maritima* cell extracts

Substrates	Activity (U/mg)	
	H_2S formation (80°C)	NADH oxidation (45°C)
NADH + $Na_2S_2O_3$	0.03 ± 0.01	0.05 ± 0.03
NADH + $Na_2S_2O_3$ + CoA	0.10 ± 0.05	0.17 ± 0.08

Table S2 Primers used for qRT-PCR

Name	Sequence
qMbxA_F	ATCTTGAGCAAGGGGAGTGC
qMbxA_R	GGTGGTGTGATGAGGTACGG
qNSR_F	GTGACGTTCTTCCCGGACTT
qNSR_R	AGCTTTTCACGAAGAGCGGA
qYeeE_F	ACTTACAGAACCGGCGAAGG
qYeeE_R	TTTCACTCCGAAGGCGTACC
qRnfC_F	AGGGCACCTCTACCTCAGAA
qRnfC_R	CCAAGGATTGGGTGCAGGAT
q16S_F	TAGCTTCAGCACGGAGGGAT
q16S_R	CCTGGTGTGTCGGATGTGAA

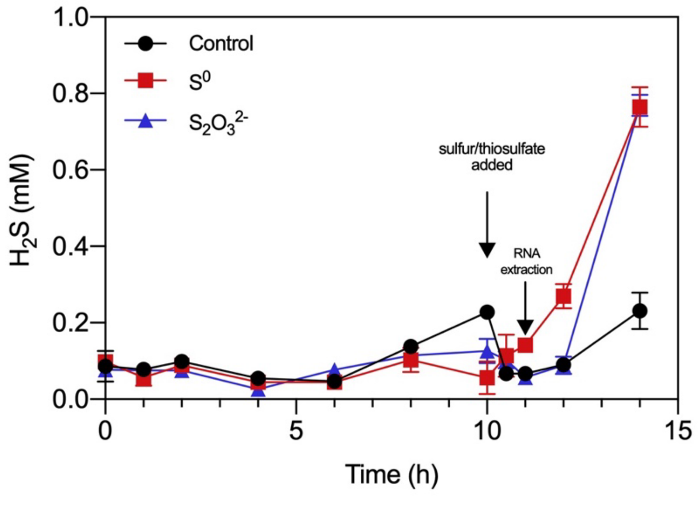

Fig S1 H_2S formation during the growth of *T. maritima*. 5 g S^0 per liter of culture or 30 mM thiosulfate was added at 10 h, and the cells were collected after 20 min and 1 h for total RNA extraction.

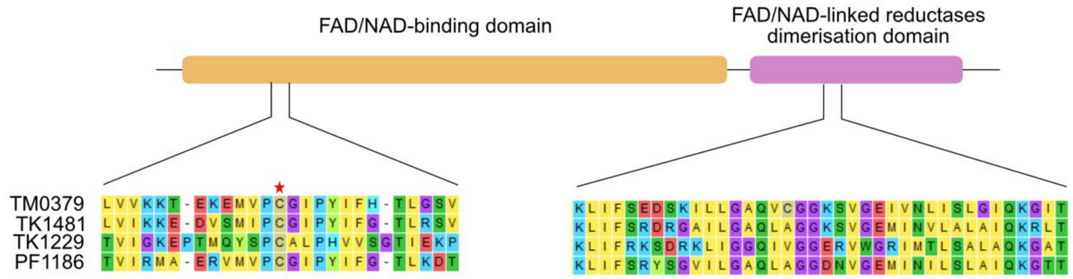

Fig S2 Conserved domains of NSR homologs. Conserved domains were analyzed by InterPro, sequence alignment was performed by MEGA 7. The cysteine labeled by red star (which is conserved in NSR homologs) was regard as an important residue for sulfur reduction (Kobori et al., 2010).

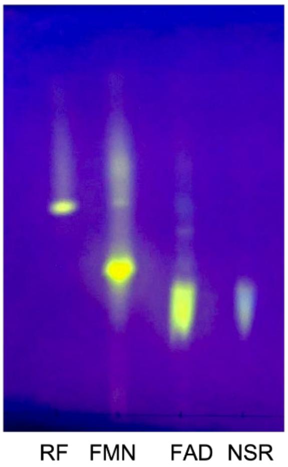

Fig S3 Thin-layer chromatography detection of flavins in NSR. NSR protein was heated at 100°C for 10 min, and proteins was removed by centrifugation. The supernatant was applied for the analysis. Authentic riboflavin (RF), FMN, and FAD were used as standards. The mobile phase was a solution of butanol-acetic acid-water (12:3:5). The fluorescence of flavin spots on TLC plates was photographed with UV illumination.

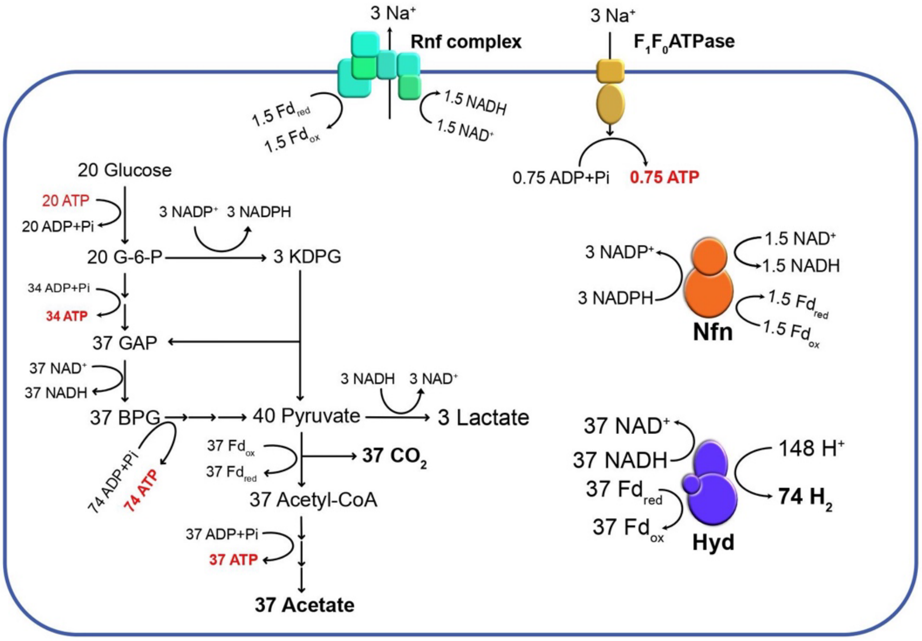

Fig S4 Proposed metabolism of *T. maritima* in the absence of S^0 or thiosulfate.

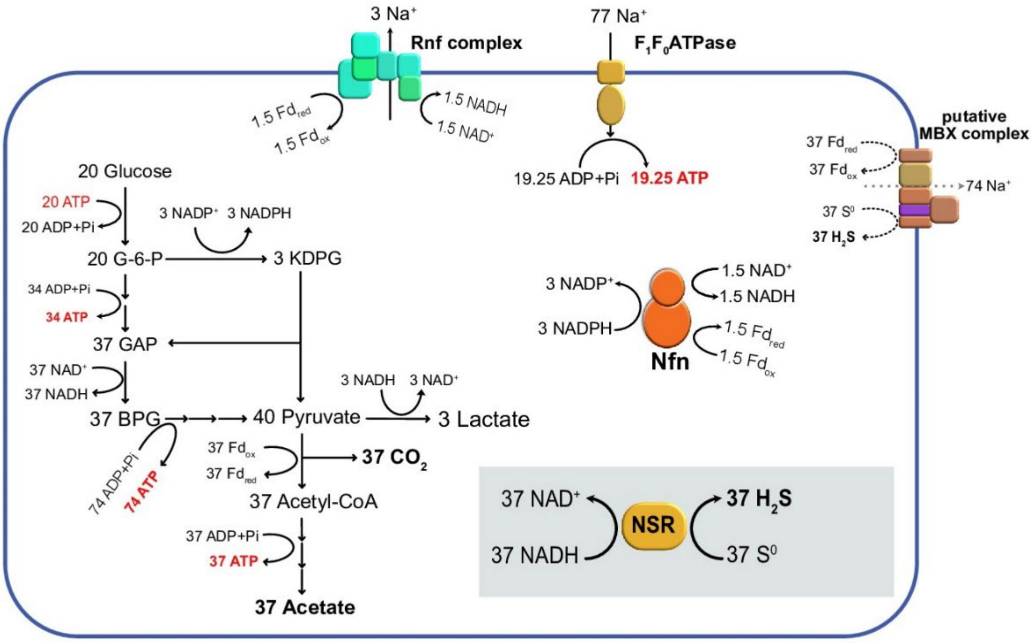

Fig S5 Proposed metabolism of *T. maritima* in the presence of S^0.

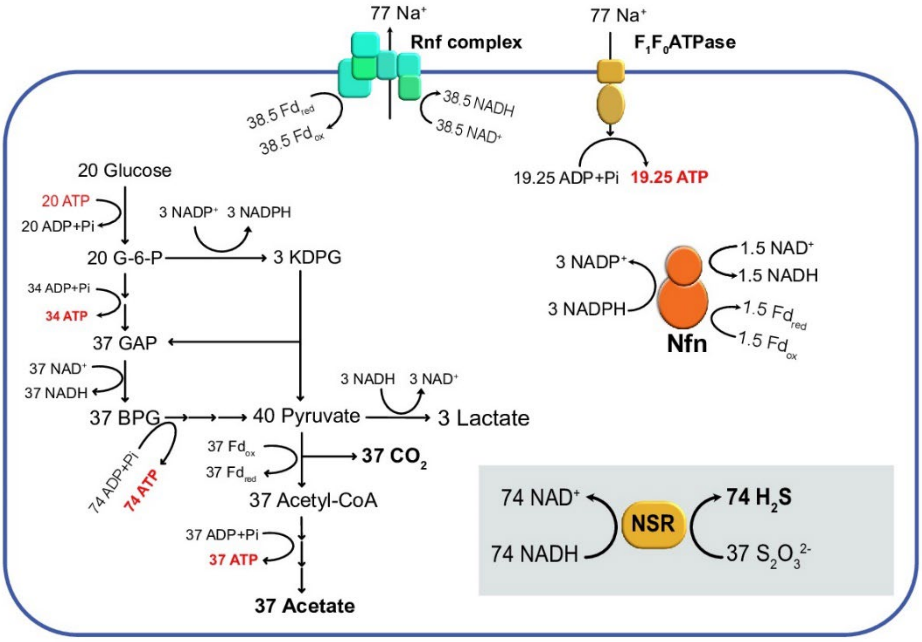

Fig S6 Proposed metabolism of *T. maritima* in the presence of thiosulfate.

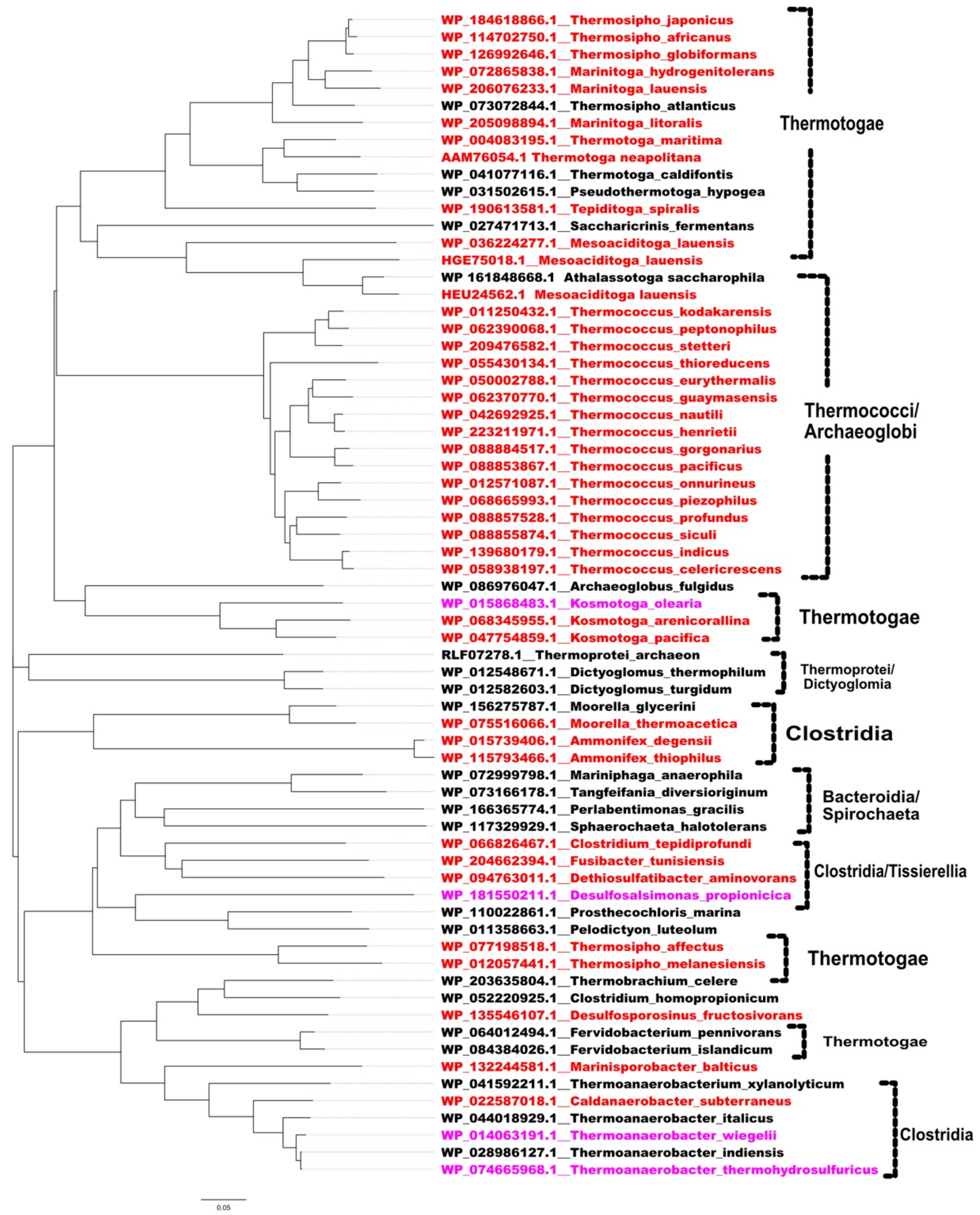

Fig S7 Neighbor-Joining tree of NSR homologs. Homologs of NSR with more than 45% identity was used to constructed neighbor-joining phylogenetic tree. Species reported with ability to reduce S^0 or thiosulfate were colored in red, and species reported to reduce thiosulfate only were colored in magenta.

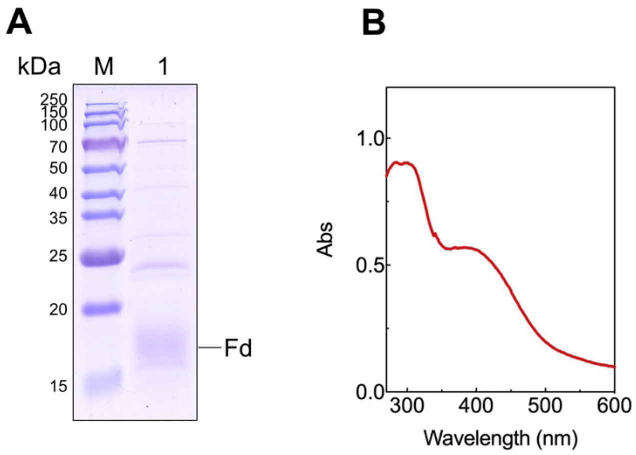

Fig S8 SDS-PAGE (A) and UV-vis absorption spectrum (B) of the recombinant *T. maritima* ferredoxin (Fd).

Reference:

Kobori H, Ogino M, Orita I, Nakamura S, Imanaka T, Fukui T. 2010. Characterization of NADH oxidase/NADPH polysulfide oxidoreductase and its unexpected participation in oxygen sensitivity in an anaerobic hyperthermophilic archaeon. J Bacteriol 192:5192–5202.
